# Laparoscopic cholecystectomy for symptomatic cholecystolithiasis (CCL) in “Kasabach–Merritt syndrome” (KMS) (Kaposi-tumor like hemangioendothelioma with case-specific perioperative management)

**DOI:** 10.1515/iss-2022-0017

**Published:** 2023-08-03

**Authors:** Stephan Arndt, Cora Wex, Inken Häusler-Pliske, Dörthe Jechorek, Hardy Krause, Zuhir Halloul, Frank Meyer

**Affiliations:** Department of General, Abdominal, Vascular and Transplant Surgery, University Hospital, Magdeburg, Germany; Institute of Pathology, University Hospital, Magdeburg, Germany; Division of Pediatric Surgery, Department of General, Abdominal, Vascular and Transplant Surgery, University Hospital, Magdeburg, Germany; Division of Vascular Surgery, Department of General, Abdominal, Vascular and Transplant Surgery, University Hospital, Magdeburg, Germany

**Keywords:** cholecystectomy, elective general surgery, giant hemangioma, hemangioendothelioma, Kasabach-Merritt syndrome (KMS), vascular surgery

## Abstract

**Objectives:**

The Kasabach–Merritt syndrome (KMS) is characterized by the occurrence of hemangioendothelioma (giant hemangioma with thrombosis leading to thrombocytopenia), which can be associated with disseminated intravasal coagulation. Specific aim: Based on (i) selective references from the current scientific literature and derived recommendations as well as (ii) own experiences obtained in the diagnostic and perioperative management of a representative case from daily practice in abdominal surgery, the specific case undergoing elective cholecystectomy (CCE) in KMS is to be described by means of scientific case report.

**Case presentation:**

(Patient-, finding- and treatment-specific characteristics): – Medical history: 72-years old female patient with a known KMS of the left arm and upper thorax, recurrent thrombophlebitis of the left arm and thoracic veins, previous upper GI bleeding (Mallory-Weiss syndrome in 2006, chronic anemia in lack of vitamin B12, type-A gastritis, former bleeding complications after teeth extraction/open appendectomy 1962/Caesarean section 1968 with need of transfusion [60 red blood cell packages]), intraabdominal adhesions, hypothyreosis, initial liver cirrhosis. – Symptomatology: Characteristic for cholecystolithiasis (CCL). – Diagnostic: Abdominal ultrasound shows CCL, fibroscan does not confirm suspicious cirrhosis. Laboratory parameters showed: Activation of intravasal coagulation with elevated prothrombin fragments, D-dimers and reduced antiplasmin concentration. Accelerated fibrinolysis capacity; currently, no secondary thrombocytopenia or factor-13 decrease. In addition, fibrinogen concentration within normal range, no hint onto the manifestation of an aquired von-Willebrand’s syndrome. – Diagnosis: Chronic fibrosing cholecystitis in CCL after former acute cholecystitis (3 months ago) with indication for surgical intervention. – Therapy: Laparoscopic CCE including careful exploration of upper abdominal cavity for KMS manifestation (with no revision of bile duct) and peritoneal adhesiolysis (histological finding, chronic fibrosing cholecystitis with thickening of the wall of the gall bladder but no hint of malignancy) under perioperative prophylaxis with antibiotics and temporary cessation of platelet medication for 7 d preoperatively, “bridging” with low molecular weight heparin (Clexane, 1 × 40 mg s.c.; Sanofi-Aventis, Frankfurt/Main, Germany); 1 h preoperatively, 15–20 mg/kg body weight Cyclocapron i.v. (once again 6–8 h postoperatively; thereafter, 500 mg of Cyclocapron 4×/d until the 3rd postoperative day). – Intraoperatively: Congestion of veins but not at the immediate surgical field (gall bladder, hepatic bed of the gall bladder, Calot’s triangle). – Outcome: Uneventful, in particular, no (bleeding) complications.

**Conclusions:**

If surgical approach is indicated, the intervention should be thoroughly planned (in particular, under elective circumstances) with regard to hemangioma site and extension as well as distance to the surgical field and possible surgical alternative options (surgical access site, open/laparoscopic approach etc.) to prevent – at the best possible rate – bleeding complications intra-/postoperatively and, thus, to provide adequate patient safety.

## Introduction

The Kasabach–Merritt syndrome (KMS; ICD 10, D18.0, D69.5) describes the occurrence of hemangioendotheliomas (giant hemangiomas) with an ongoing thrombosis within the lesion, which leads to the consumption of coagulation factors and platelets with a disseminated intravascular coagulation (DIC) [1].

The aim of this scientific case report was based on

(i) selective references from the topic-related medical scientific literature and their recommendations as well as
(ii) own clinical management experiences from daily practice in surgery, to describe the frequent elective cholecystectomy (CCE) in symptomatic cholecystolithiasis with the coincidence of a rare clinical picture of KMS.

## Case report

A 72-years old female patient was transferred with a status after previous episode of conservatively treated acute cholecystitis in cholecystolithiasis to our outpatient clinic for elective cholecystectomy. Medical history was significant for KMS of the left arm and the left upper thoracic wall with recurrent thrombophlebitis of the left arm and thoracic veins. In addition, there were numerous bleeding complications after

– dental extraction,
– open appendectomy in 1962 (and)
– Caesarean section in 1968 (in which 60 red blood cell packs had been necessarily transfused) previously.

Furthermore, medical history comprised

– status after upper GI bleeding in Mallory-Weiss syndrome in 2006,
– chronic anemia in vitamine-B12 lack,
– type-A gastritis,
– hypothyreosis,
– arterial hypertonus,
– initial stage of liver cirrhosis,
– chronic renal insufficiency – stage II (as well as)
– allergy against macrolide and penicillin antibiotics.

The medication prescribed by the family practitioner comprised L-thyroxin, an ACE inhibitor, hydrochlorothiazide and clopidogrel (platelet aggregation inhibitor) to avoid exaggerated platelet aggregation. The patient was provided with a compression sock for the left arm.

Due to the symptomatic cholecystolithiasis after an episode of an acute cholecystitis, indication for an elective laparoscopic cholecystectomy was derived.

**Figure 1:**
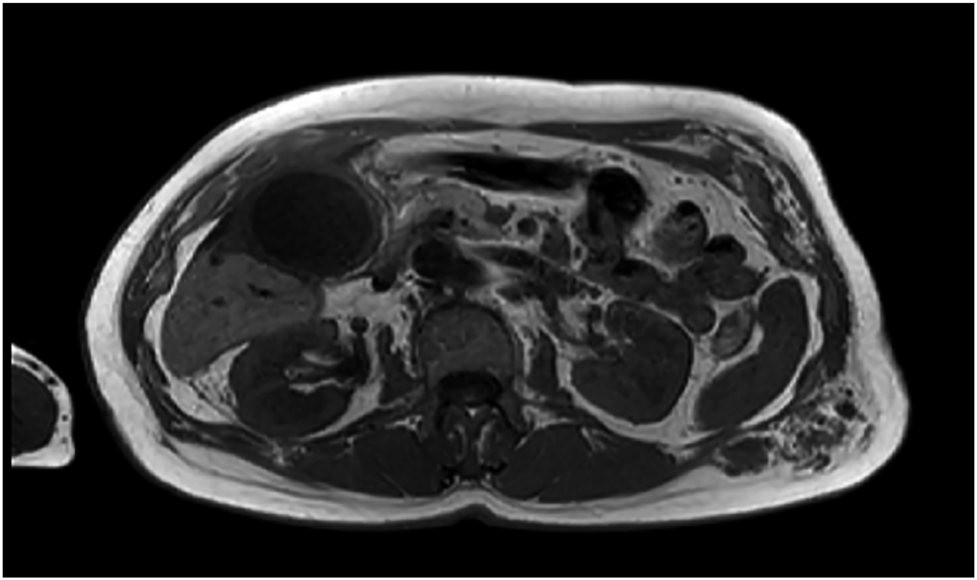
Angio-MRI (transversal scan of the upper abdomen – gall bladder: In addition to the enlarged gall bladder [hydrops]) including a thickened wall → hemangioendothelioma at the left ventral and dorsal wall of the trunk.

At the regional hospital, angio-MRI (Figures 1 [Fig j_iss-2022-0017_fig_002] [Fig j_iss-2022-0017_fig_003]–4) of the upper abdomen including MRCP was performed, which revealed cholecystitis in cholecystolithiasis and commencing liver cirrhosis. As part of the preoperative work-up, echocardiography was performed, by which heart insufficiency due to volume load by arteriovenous shunts caused by KMS and “Cirrhose cardiaque” could be definitely excluded. As marker of an intravasal activation of coagulation, increased prothrombin fragments, D-dimers and a decreased antiplasmin concentration as well as increased capacity of fibrinolysis were detected. A secondary thrombocytopenia or a decline of fibrinogen and factor XIII as well as an acquired von-Willebrand’s syndrome were excluded.

**Figure 2: j_iss-2022-0017_fig_002:**
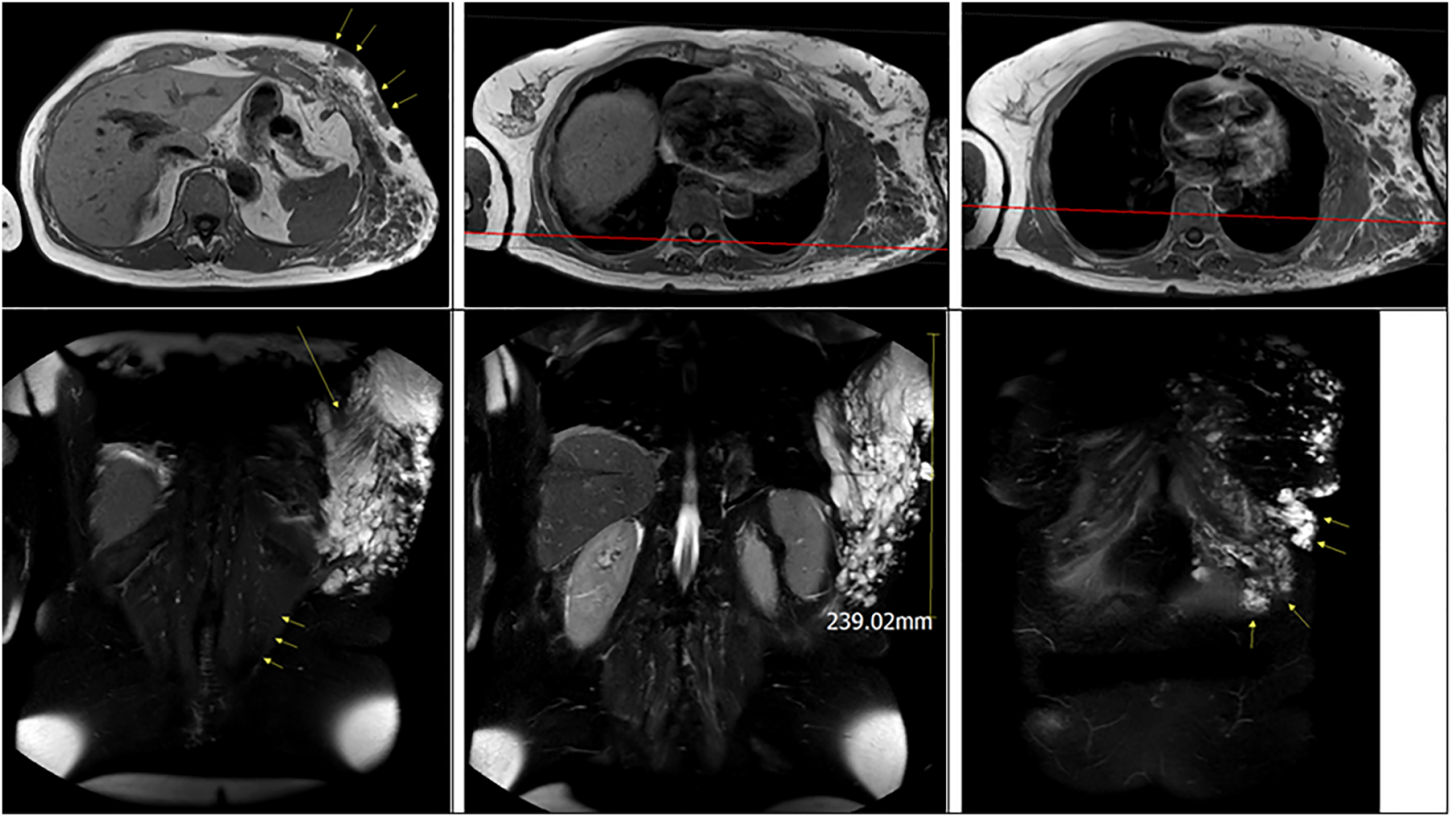
MRI: (A) T1 axial scan – cutaneous site at the arcus; (B) T1 axial scan – finding at the M. latissimus dorsi; (C) T1 axial scan – main finding at the posterior axillar line; (D) T2 coronary scan – M.-latissimus-dorsi line; (E) T2 coronary scan – main finding at the posterior axillar line; (F) T2 coronary scan – cutaneous site at the arcus.

**Figure 3: j_iss-2022-0017_fig_003:**
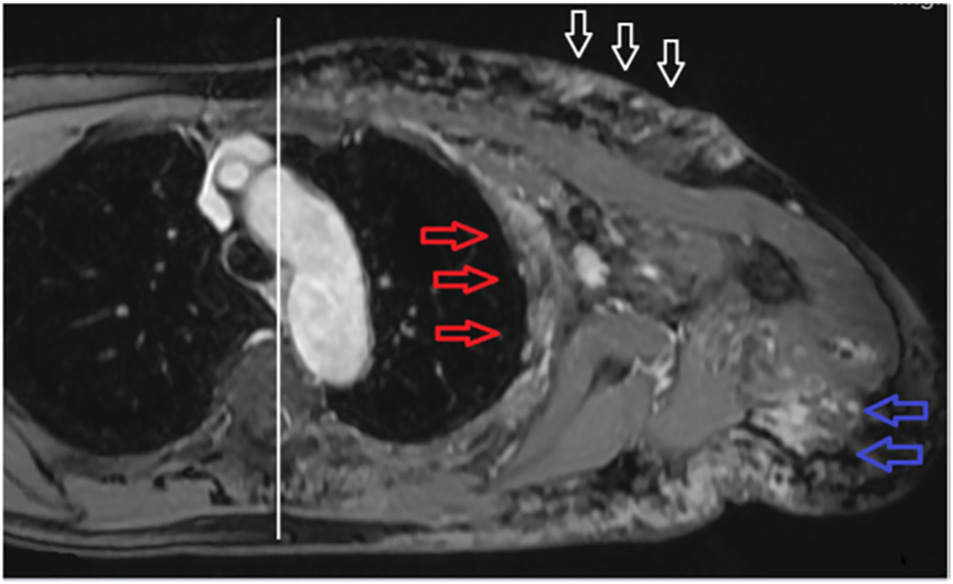
Contrast media (CM)-enhanced fat-suppressed axial T1 sequence: Tubular-granular CM-enriched findings including intercostal musculature (red arrows), musculature of the upper arm (blue arrows) and of the cutis (white arrows). Limitation of the malformation to only one quadrant of the trunk (see white middle line).

**Figure 4:**
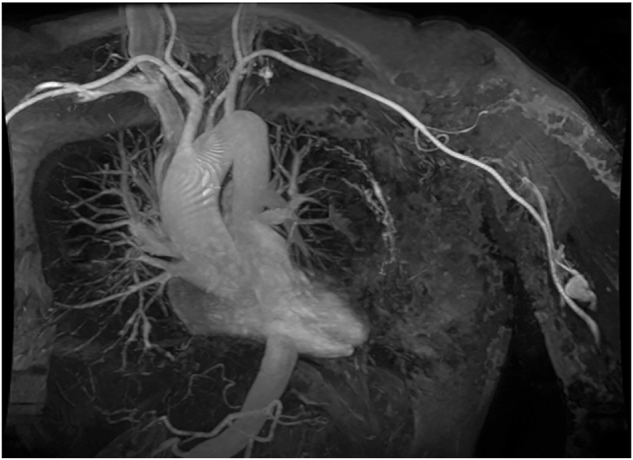
MR angiography (coronary scan) – pronounced intercostal arteries.

Hemostaseological consultation recommended pausing platelet inhibition over 7 d by means of “bridging” with low molecular heparin and inhibition of hyperfibrinolysis with drugs (tranexamic acid) until the 3rd postoperative day or depending on symptomatology and hemostaseologic monitoring. For perioperative antibiotic prophylaxis, Imipenem was used.

**Figure 5:**
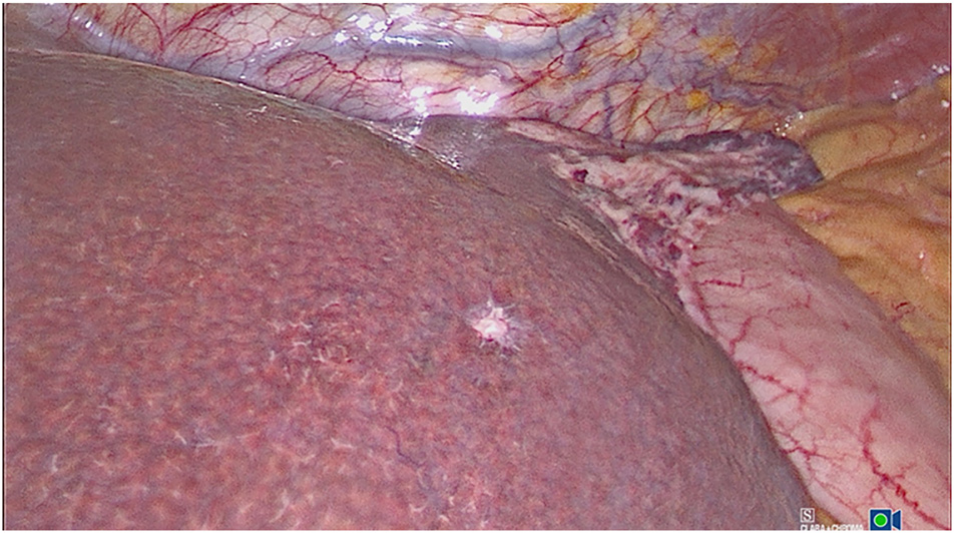
Intraoperative laparoscopic finding of a hemangioendothelioma of the left wall of the trunk and left triangular ligament. Additional finding: Small von-Meyenburg complex at the junction of the hepatic segments II and III in liver cirrhosis.

**Figure 6:**
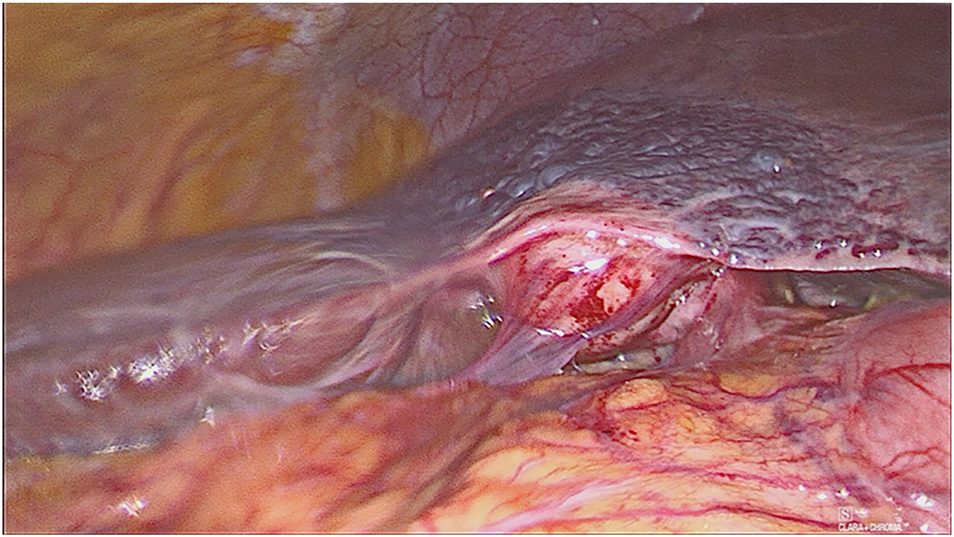
Intraoperative laparoscopic finding of a partially chronic, partially acute cholecystitis as well as venous malformation of the liver (hepatic hemangioma) with parenchymatous transition of small nodules indicating liver cirrhosis.

The camera trocar was placed in the perumbilical region according to the left-sided manifestation of the vascular malformation known from the angio-MRI (Figures 1 [Fig j_iss-2022-0017_fig_002] [Fig j_iss-2022-0017_fig_003]–4). Under diaphanoscopic control (Figure 5), two further epigastric working trocars were placed slightly more to the right side. Intraoperatively (Figure 6), gall bladder was characterized by acute as well as chronic inflammation aspects. The liver showed macroscopically venous malformations and a commencing parenchymatous alteration with small nodules. Laparoscopic cholecystectomy was performed with no complications; placement of drainage was renounced.

**Figure 7:**
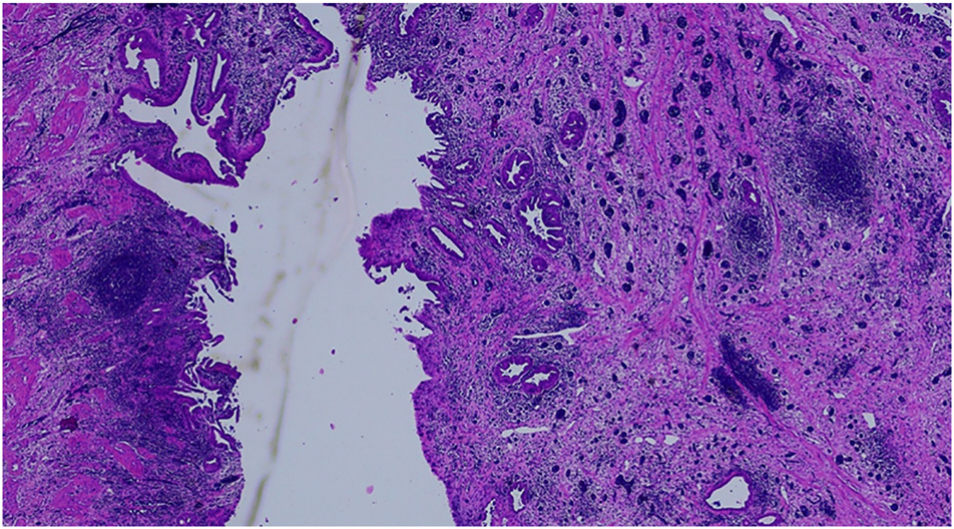
Magnification (×20) of the fundus of the gall bladder. Overview, vessels congested with blood [1] and chronic inflammatory infiltrate [2].

Histopathological investigation (Figure 7) revealed chronic fibrosing cholecystitis with thickening of the wall with no hint for malignancy.

Patient’s further clinical course was uneventful – she could be discharged with subjective well-being, no wound problems and excluded excessive activation of coagulation as well as stable hemogram on the 3rd postoperative day.

## Discussion

KMS is characterized by a combination of (giant) hemangiomas of the skin or/and inner organs with the potential for a DIC. In this context, genetic cause is assumed, however, the exact cause is not known. Predominatly, women are affected with a prevalence of 0.7/100,000 [2] (mortality, 30 % [3, 4]). Median age of disease onset is 2 months, rarely, it manifests in adult age (Table 1).

**Table 1:** Diagnostic of the Kasabach–Merritt syndrome – possible criteria: DIC score of the ISTH for diagnosis-finding of a manifest disseminated intravasal coagulation.

	Values	Points
PT/INR^a3^	PT normal^a1^ PT prolonged 3–6 s^a2^ PT prolonged > 6 s^a2^	0 1 1
Platelet count	> 100/nL 50–100/nL < 50/nL	0 1 2
Fibrin marker (D-Dimer)	Normal Slightly increased Strongly increased	0 1 2
Fibrinogen	> 1 g/L < 1 g/L	1

^a^1 according to INR <1.25; ^a^2 according to INR 1.25–1.7; ^a^3 according to INR >1.7; DIC, “disseminated intravascular coagulation”; ISTH, “International Society for Thrombosis and Hemostasis”; PT, prothrombin time. → DIC in case of >5 points.

***Symptomatology*** is predominated by the giant hemangiomas preferentially distributed at the extremities, in particular, by a bleeding tendency due to platelet consumption in disseminated intravascular coagulation and possibly reaching to a threatening multi-organ failure.

***Diagnosis-finding*** is not possible by direct detection (exact cause unknown) but the symptom combination is considered unique (congenital hemangiomas, thrombocytopenia, DIC possible). Usually, the disease does not progress, spontaneous healing is considered to be controversial.

The therapeutic spectrum comprises:

– laser-based surgical removal/excision,
– embolization (interventional radiology):
– * risk of local necroses,
– * tumour lesions (hemangiomas) do have supplying vessels;
– external compression,
– α-Interferons and corticosteroids:
– * mitigate particularly the high propensity of recurrency;
– Propanolol – mostly not sufficient as the only medication (or)
– substitution of platelets only in bleeding tendency due to thrombocytopenia.

The therapeutic success is assessed by recovery of hemostasis and elimination of tumor cells. In this context, the early definitive surgical therapeutic success of KMS results in a higher healing rate and lower rate of drug-induced adverse effects.

***Histopathological finding*** revealed a locally aggressive, severely varying cell growth involving several tissue layers (in contrast, in case of “normal” hemangioma, only one layer is affected). Both entities are positive for.

– D2-40,
– LYVE1 (and)
– Prox-1 but negative for GLUT-1.

Skin hemangiomas are associated with a mortality of 10 %, retroperitoneal tumor lesions of this entity – depending on the reference – up to 60 %. It has to be taken into account that this hemangioma site can be overlooked in physical examen in case of only mild complaints, by which mean mortality of 30 % can increase.

Considering differential diagnosis, there are hemangiomas, which can occur in 3–5 % of infants and which can be classified as embryonal “tumor lesion” with endothelial proliferation in secondary formation of vascular lumens. Eighty % of the hemangiomas reduce in size until the 5th year of life. Histopathologically, blood-filled wide vessels with flat endothelial lining are characteristic, smaller hemangiomas are also treatable with propanolol.

The presented case demonstrates representatively remarkable aspects of surgical decision-making with regard to an operation:

In case of an indicated surgical intervention, the approach needs to be thoroughly planned (in particular, in an elective setting) with regard to the hemangioma extension and the fact that the lesions can possibly affect the potential surgical field as well as to verify alternative options of the approach (access site, open/laparoscopic surgery etc.) – finally to avoid intra-/postoperative bleeding complications as good as possible and, thus, to maintain a high patient safety.

As limitation, only experience of a single case related to the diagnostic and therapeutic, in particular, perioperative management could be obtained.

## Resumé

Surgical diseases with coincidental KMS require close collaboration with a hemostaseologist and adequate preoperative diagnostic of coagulation status as well as appropriate perioperative management.

Surgical intervention as well as to avoid severe bleeding under perioperative thrombosis prophylaxis and inhibition of hyperfibrinolysis with drugs (Tranexamsäure) prevent activation of the coagulation system and consumption of coagulation factors. Even a platelet transfusion is to be prevented due to, thus, a possible activation of coagulation system.

In particular, elective setting allows to clarify the hemangioma extension and whether it is tangent to the potential surgical field (using ultrasound, angio-CT scan, MRI) to derive reasonable modifications of the approach (access site, open/laparoscopic surgery etc.).

In addition,

– coincidental heart insufficiency due to high shunt volumes,
– presence of a “Cirrhose cardiaque” (and)
– infectious diseases due to massive transfusions
– should be taken into account in planning elective surgical care.

## Supplementary Material

Supplementary Material
