# Supplementary material for: Laparoscopic cholecystectomy for symptomatic cholecystolithiasis (CCL) in “Kasabach–Merritt syndrome” (KMS) (Kaposi-tumor like hemangioendothelioma with case-specific perioperative management)
Source: Innov Surg Sci. 2023 Aug 3;8(2):113–7. doi: 10.1515/iss-2022-0017 (PMC10696942; doi:10.1515/iss-2022-0017)
Supplement: Supplementary file 1 — Supplementary Material [file j_iss-2022-0017_suppl_001.pdf]

Reviewer Assessment

S. Arndt et al.: Laparoscopic cholecystectomy for symptomatic cholecystolithiasis (CCL) in “Kasabach-Merritt syndrome” (KMS)

Comments to Original Submission

Reviewer 1: anonymous

Date received: 16-Feb-2023
Reviewer recommendation: **Reject for the following reasons**
Reviewer overall scoring: **High**

Comments to author:
Dear Madam, dear Sir

This is a very interesting article about the specifics of a high risk cholecystectomy.
The specific intraoperative and postoperative risks were clearly presented. In addition, the special preoperative management was explained.
In the following, the case was adequately discussed scientifically. In addition, the limitations of the study were presented.

Reviewer 2: Franzke, Timm

Date received: 27-Mar-2023
Reviewer recommendation: **Accept in present form**
Reviewer overall scoring: **High**

Comments to author:
This case report may be published as is

Reviewer 3: anonymous

Date received: 27-Mar-2023
Reviewer recommendation: **Accept in present form**
Reviewer overall scoring: **High**

Comments to author:
Interesting case report which shows the need for adequate preoperative diagnostics and perioperative management before surgical intervention in case of relevant comorbidities as well as rare coincidental diseases.

Comments by the Editorial Office to the Editor-in-Chief Decision ‘Accept’

The comments of the first reviewer were opposing the recommendation which is why additional reviewers were invited.
